# Rutaecarpine Aggravates Acetaminophen-Induced Acute Liver Injury by Inducing CYP1A2

**DOI:** 10.3390/toxics12070515

**Published:** 2024-07-18

**Authors:** Meiqi Wan, Hua Gao, Xiaoyan Liu, Youbo Zhang

**Affiliations:** 1 State Key Laboratory of Natural and Biomimetic Drugs, Key Laboratory of State Administration of Traditional Chinese Medicine for Compatibility Toxicology, Department of Natural Medicines, School of Pharmaceutical Sciences, Peking University, Beijing 100191, China; wanmeiqi2018@163.com (M.W.); gaohua_cpu@163.com (H.G.); 2 Laboratory of Metabolism, Center for Cancer Research, National Cancer Institute, National Institutes of Health, Bethesda, MD 20892, USA; 3 Henan Engineering Research Center of Medicinal and Edible Chinese Medicine Technology, Henan University of Chinese Medicine, Zhengzhou 450046, China

**Keywords:** rutaecarpine, acetaminophen, hepatotoxicity, CYP1A2, inflammatory cytokines

## Abstract

In this study, we investigated whether rutaecarpine could aggravate acetaminophen-induced acute liver damage in vivo and in vitro. CCK-8 and apoptosis assays were performed to verify the cytotoxicity of acetaminophen to L02 cells with or without rutaecarpine. The expression levels of the target proteins and genes were determined using Western blotting and qRT-PCR. The liver pathological changes were evaluated with hematoxylin and eosin staining, while the aspartate aminotransferase (AST) and alanine aminotransferase (AST) levels in plasma were measured to assess the liver damage. Our results revealed that pretreatment of the cell and mice with rutaecarpine significantly aggravated the acetaminophen-induced liver damage. Mechanistically, rutaecarpine induces the CYP1A2 protein, which accelerates the metabolism of acetaminophen to produce a toxic intermediate, N-acetyl-p-benzoquinone imine (NAPQI), leading to severe liver inflammation. Rutaecarpine exacerbated the liver damage by upregulating CYP1A2 and proinflammatory factors. These findings highlight the importance of carefully considering the dosage of rutaecarpine when combined with acetaminophen in drug design and preclinical trials.

## 1. Introduction

In recent years, the combination therapy of traditional Chinese medicine (TCM) and synthetic drugs has become increasingly common in clinical settings for the treatment of various diseases. Due to the diversity and complexity of the compounds in TCM, particular and ongoing attention should be paid to their side effects and safety when used in combination with synthetic drugs. The liver, which is rich in drug-metabolizing enzymes, is the primary organ responsible for many critical physiological functions, including drug metabolism and detoxification. However, some drugs can lead to hepatotoxicity, causing liver damage or acute and chronic liver failure, resulting in higher clinical drug toxicity-related incidence and mortality rates. As the prevalence of chemical drug use rises, there is growing concern over the escalating number of adverse reactions resulting from the concurrent use of Chinese and Western medications. Therefore, conducting research in this area is imperative.

An overdose of acetaminophen (APAP) is a major cause of acute liver failure (ALF) [1,2,3]. Some reports indicated that therapeutic doses of APAP can increase serum transaminases for a few days [4,5]. N-acetyl-p-benzoquinone imine (NAPQI) is a toxic intermediate and the main product of APAP via oxidation by cytochrome P450 enzymes (CYP2E1, CYP3A11 and CYP1A2). High production of NAPQI in the liver causes excessive depletion of glutathione (GSH) and production of NAPQI–protein adducts, ultimately leading to mitochondrial oxidative stress and acute liver inflammation [6,7,8].

Euodiae fructus (EF) is the dried unripe fruit of Euodia rutaecarpa (Juss.) Benth., Euodia rutaecarpa (Juss.) Benth. var. officinalis (Dode) Huang and Euodia rutaecarpa (Juss.) Benth. var. bodinieri (Dode) Huang. It has been used for centuries to treat gastrointestinal diseases, thermo-regulatory issues and headache in clinical settings [9,10]. However, the potentially hepatotoxic side effects of EF have been consistently reported [11,12,13]. Zhang et al. [14] found that the metabolic intermediate might contribute to the toxicity of the EF extract in L02 cells. The hepatotoxicity of the water extract was higher than that of the ethanol extract and volatile oil of EF, with all forms exhibiting dose-dependent toxicity in rats [15]. The clinical hepatotoxicity of EF is usually caused by consuming unprocessed materials or overdosing, with the potential mechanisms involving the activation or upregulation of inflammatory factors, mitochondrial injury and peroxidation damage [16]. Phytochemical and pharmacological evaluations have shown that indolo-quinazoline alkaloids, such as rutaecarpine (Rut) and evodiamine (Evo), and quinolone alkaloids, such as 1-methyl-2-nonyl-4(1H)-quinolone (Mnq), 1-methyl-2-undecyl-4(1H)-quinolone (Muq), and evocarpine (Evc), are the main bioactive ingredients of EF [12,17,18,19]. 

Some studies have suggested that CYP3A4 plays a role in the metabolic activation and dehydrogenation of Evo and Rut, leading to potential liver toxicities in humans through the formation of electrophilic intermediates [20,21]. 

The impact of Rut on hepatotoxicity remains uncertain, while some evidence indicates that Rut may protect against acetaminophen-induced liver damage by enhancing Nrf2-mediated antioxidant enzyme activity [22]. However, other reports suggest that Rut could worsen drug-induced liver injury, such as acetaminophen-induced hepatotoxicity, by altering the acetaminophen pharmacokinetics [23], inhibiting CYP activity [24], and increasing the levels of AST and ALT in mouse serum through regulation of CYP3A4 [20,25]. This study aims to investigate whether Rut exacerbates acetaminophen-induced DILI by establishing in vivo and in vitro liver damage models. The findings seek to clarify the potential hepatotoxic effects of Rut in combination with acetaminophen and provide insights into the safety of using Chinese and Western medicines together.

## 2. Materials and Methods

### 2.1. Chemicals and Reagents

**Figure 1:**
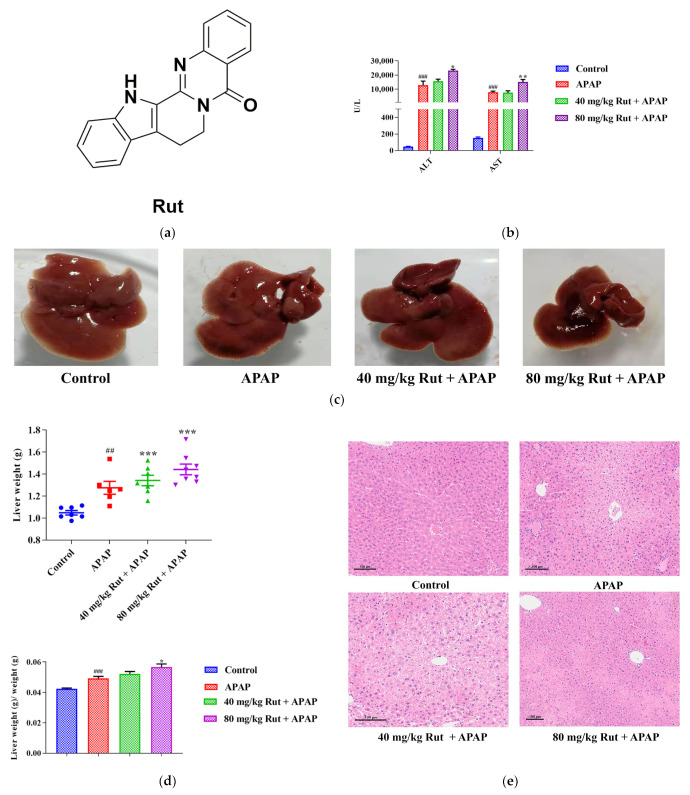
Effects of APAP and APAP + Rut on liver damage in mouse liver. (**a**) The chemical structure of Rut. (**b**) The activities of ALT and AST in mouse serum, data are presented as mean ± SEM (*n* = 5), statistical difference: compared with control, ^###^ *p* < 0.001; with APAP group, * *p* < 0.05 and ** *p* < 0.01. (**c**) Morphological changes in mouse liver. (**d**) The liver index of mouse, data are presented as mean ± SEM (*n* = 5), statistical difference: compared with control, ^##^ *p* < 0.01, ^###^ *p* < 0.001; with APAP group * *p* < 0.05, *** *p* < 0.001. (**e**) H&E staining of mouse liver, original scale: 10×. Rut pretreatment exacerbated liver injury.

Rut was isolated in our lab with a purity > 98%, and the structure was identified by NMR and high-resolution mass spectrometry (Figure 1a). Acetaminophen (APAP) and α-Naphthoflavone were provided by Sigma Chemical Co. (St. Louis, MO, USA). TRIzol reagent was obtained from Thermo-Fisher Scientific (Halethorpe, MD). Moreover, 5×qRT Super Mix and Mon-Amp TM SYBR Green qPCR mix (Low ROX) were purchased from Monad (Suzhou, Jiangsu, China). RIPA Lysis Buffer was provided by Beyotime (Shanghai, China). RPMI-1640 cell culture medium and fetal bovine serum (FBS) were obtained from Gibco-BRL (Grand Island, NY, USA). Anti-cytochrome P450 1A2 (ab22717) and anti-β-actin (ab8227) were obtained from Abcam (Cambridge, Cambridgeshire, UK). The assay kit for apoptosis and the Cell Counting Kit-8 (CCK-8) were purchased from Meilunbio (Dalian, China).

### 2.2. Culture of Cell Lines 

The human normal liver cell line L02 was provided by Beijing University of Chinese Medicine. The cells were cultivated in a humidified atmosphere in 5% CO_2_ at 37 °C in RPMI-1640 (containing 10% FBS and 1% penicillin/streptomycin). 

### 2.3. Cell Viability Assay

The cytotoxicity of Rut and APAP was measured by the CCK-8 kit. Briefly, cells were seeded into 96-well plates (8 × 10^4^ cells/mL) in a CO_2_ incubator at 37 °C for 24 h, then treated with different concentrations of Rut (20, 10, 5, 2.5, 1.25 and 0.625 μM) for 18 h. A range of concentrations of APAP (50, 25, 12.5, 6.25, 3.125 and 1.5625 mM) were subsequently added to the medium and treated for 6 h at 37 °C. Afterwards, the CCK-8 solution was added to each well with 10% of the original concentration and co-incubated for 4 h. The optical density (OD) value was measured at 450 nm using a microplate reader (Thermo Fisher Scientific, Waltham, MA, USA).

### 2.4. Apoptosis Assay

Briefly, L02 cells were seeded into 12-well plates with a density of 2.5 × 10^5^ cells/mL and cultured for 24 h. The control group, model group (30 mM APAP), and two concentrations of Rut groups (2.5 μM and 10 μM) were set, respectively, with three replicate wells for each group. The cell suspensions were treated with the protocol for the apoptosis detection kit and detected by flow cytometry (Beckman Coulter, Fullerton, CA, USA). 

### 2.5. Animal Studies

Male C57BL/6J (8 weeks old) mice were purchased from the Laboratory Animal Center of Peking University Health Science Center (Beijing, China). All the animal experiments were approved by the Biomedical Ethical Committee of Peking. The mice were housed in a specific pathogen-free environment controlled for temperature, light, and humidity (25 °C, 12-h light/dark cycle, 45–65%) for 1 week before the experiments.

Experiment 1: thirty-two mice were randomly divided into four groups (blank, APAP, 40 mg/kg Rut + APAP and 80 mg/kg Rut + APAP groups, *n* = 8). Rut was dissolved in corn oil and APAP was freshly dissolved in warm saline. The mice were administered 40 mg/kg Rut, 80 mg/kg Rut and corn oil by gavage once a day for 4 days (blank and APAP group were treated with corn oil), and treated with APAP (300 mg/kg) by intraperitoneal administration 1 h after the last drug treatment to induce hepatotoxicity (the blank group was administrated with saline). Finally, the mice were sacrificed by cervical dislocation and dissected after 24 h and a proportion of the liver tissues were fixed in 10% formalin solution, and the serum and residual liver tissues were stored at −80 °C for further use, respectively. 

Experiment 2: twenty-five male mice were randomly divided into five groups (*n* = 5). The blank, APAP and 80 mg/kg Rut + APAP groups were processed in the same way as in experiment 1. Mice in the 80 mg/kg Rut + inhibitor (α-Naphthoflavone) + APAP group and inhibitor group were separately pretreated with 80 mg/kg Rut or corn oil by gavage for 4 consecutive days. At the last day after administration, the mice were given 120 mg/kg α-Naphthoflavone by gavage one hour later, and then injected intraperitoneally with APAP or saline, respectively. The collection of serum and liver samples was consistent with experiment 1.

### 2.6. Quantitative Real-Time Polymerase Chain Reaction (qRT-PCR) 

The total RNA was reverse transcribed with reverse transcriptase based on the protocol provided with the kit. The primer sequences are shown in Supplementary Tables S1 and S2. Mon-Amp TM SYBR Green qPCR mix (Low ROX) was used for the qRT-PCR. The Ct values were compared with β-actin/GAPDH to calculate the relative gene expression levels.

### 2.7. Western Blot

The lysis of the liver tissues took place with RIPA lysis buffer (phosphatase and protease inhibitors added) to obtain the total proteins. A BCA Protein Assay Kit was applied for the protein quantification. After electrophoresis on the 10% SDS–PAGE gels, the proteins were transferred to a polyvinylidene difluoride (PVDF) membrane and blocked with 5% non-fat milk. The PVDF membrane were incubated with primary antibodies overnight at 4 °C on an orbital shaker with gentle shaking. After rinsing with TBST, the PVDF membrane was incubated with fluorescent secondary antibody (in 5% skim milk) for 1 h and rinsed with TBS three times. Finally, the Odyssey^®^ CLx Infrared Imaging System (Gene Company Limited, LI-COR, Lincoln, NE, USA) was adopted to capture and quantify the protein bands.

### 2.8. Serum Aminotransferase Analysis and Histological Examination

The ALT and AST contents of the serum were tested by an automatic biochemical analyzer. H&E staining was executed for the formalin-fixed liver tissues (embedded in paraffin and 5 μm thick sections).

### 2.9. Statistical Analysis

In this research, the GraphPad Prism 7.0 software (GraphPad Software, San Diego, CA, USA) and SPSS 26 software (IBM SPSS Inc., Chicago, IL, USA) were the main tools used for data analysis. The data in the experiments are presented as the mean ± SEM. A two-tailed Student’s *t*-test analyzed the differences between two groups, and a one-way ANOVA was used among multiple comparisons. A *p* value < 0.05 was considered to be statistically significant.

## 3. Results

### 3.1. Rut Aggravated the APAP-Induced Acute Liver Injury in Mice

#### 3.1.1. Serum Transaminases, Hepatic Index and Histological Analysis

In this study, the serum transaminases, hepatic index and histological analysis were used to evaluate the level of liver damage in mice. The ALT and AST concentration changes in the serum showed that a single dose of APAP (300 mg/kg) exposure significantly increased the levels of both transaminases compared with to the blank group (*p* < 0.001), indicating the severe hepatotoxicity of APAP. Additionally, 80 mg/kg Rut (80 mg/kg Rut + APAP) remarkably increased the concentration levels of ALT and AST when administered to mice in combination with APAP (*p* < 0.05 and 0.01, respectively) (Figure 1b), while there was no significant difference between the 40 mg/kg Rut + APAP and APAP groups (*p* > 0.05). The liver tissue of the mice in the APAP group appeared turgid with a dark color, and the liver was further aggravated in the Rut-pretreated mice (Figure 1c). The liver weight and hepatic index were more elevated in the APAP group compared to the blank group (*p* < 0.01 and 0.001, respectively) and Rut (80 mg/kg Rut + APAP) could increase these changes significantly (*p* < 0.001 and 0.05, respectively) (Figure 1d), but the hepatic index of the mice had no significant changes in the 40 mg/kg + APAP group compared to that in APAP group. The H&E staining results revealed that livers from the APAP-treated mice exhibited extensive focal necrosis and cell membrane damage, and Rut pretreatment exacerbated these symptoms (Figure 1e). These results demonstrated that Rut could aggravate APAP-induced acute liver injury in a dose-dependent manner.

#### 3.1.2. The Involvement of Cyp1a2 in Rut-Aggravated APAP-Induced Hepatotoxicity

NAPQI is a highly reactive metabolite of APAP, catalyzed by cytochrome P450. The overproduction of NAPQI depletes glutathione (GSH), leading to the formation of NAPQI-GSH adducts, which triggers mitochondrial oxidative stress and ultimately initiates liver injury. The 3-alkylindole moiety in Rut is dehydrogenated to an electrophile, 3-methyleneindolenine, which is catalyzed by cytochrome P450, and this compound subsequently binds with hepatic GSH to form 3-methyleneindolenine-GSH, causing hepatotoxicity. 

**Figure 2:**
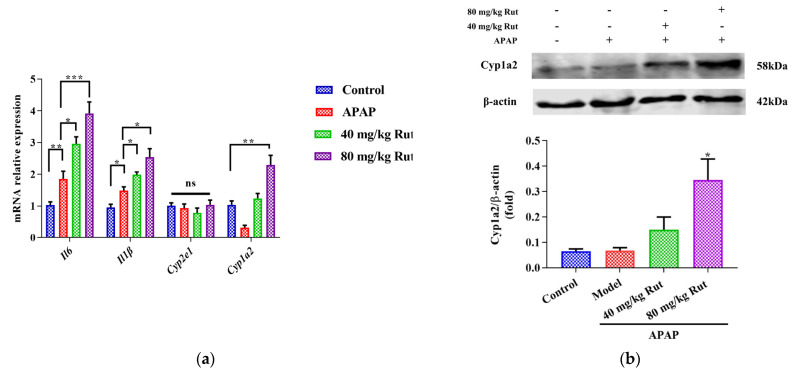
Effects of APAP and APAP + Rut on mRNA and protein levels in mouse liver. (**a**) The mRNA expression of Cyp2e1, Cyp1a2, mIl6 and mIl-1β, data are presented as mean ± SEM (*n* = 5), statistical difference: ns (no significant difference), * *p* < 0.05, ** *p* < 0.01 and *** *p* < 0.001. (**b**) Protein level of Cyp1a2, results are indicated as means ± SEM (*n* = 3), significantly different compared with control, * *p* < 0.05.

To evaluate the effect of Rut on APAP-mediated hepatic damage, we examined the expression of Cyp2e1, Cyp1a2, and pro-inflammatory cytokines. The qRT-PCR results showed that APAP significantly induced hepatic mRNA expression of Il6 and Il-1β, indicating the presence of a inflammatory reaction in the mice liver. Rut pretreatment aggravated the APAP-mediated liver toxicity in a dose-dependent manner (Figure 2a). Additionally, the mRNA and protein expression levels of Cyp1a2 in the Rut-pretreated groups were significantly upregulated compared to the APAP groups, respectively (Figure 2a,b), while there was no significant change in the Cyp2e1 mRNA expression (Figure 2a). These results demonstrated that Rut increased the inflammatory response in mice administered APAP. The potential mechanism may involve the modulation of CYP1A2 expression.

### 3.2. CYP1A2 Inhibitor Reversed Rut Aggravation of APAP-Induced Hepatotoxicity

#### 3.2.1. The Serum ALT and AST Levels and H&E Staining

**Figure 3:**
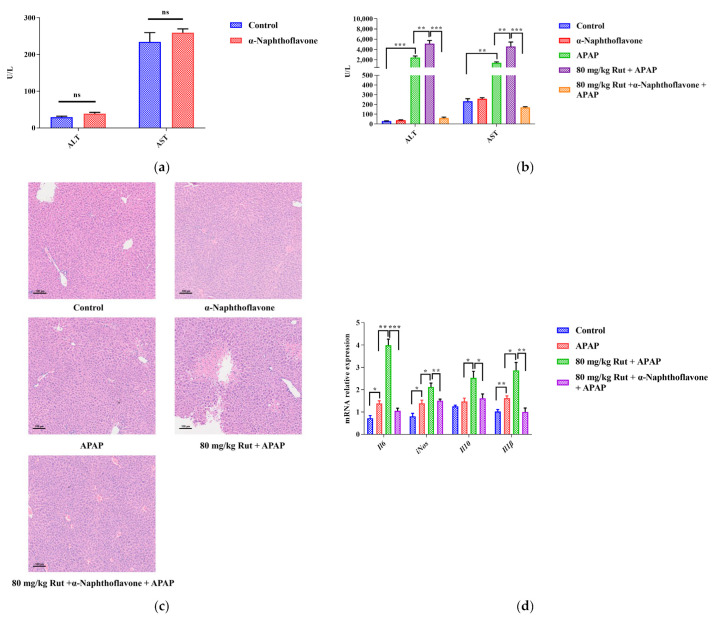
α-Naphthoflavone reversed the effects of Rut-aggravated APAP-induced acute liver injury. (**a**) The ALT and AST levels in mouse serum after treatment with α-Naphthoflavone. ns (no significant difference). (**b**) The ALT and AST levels in APAP/Rut-induced mouse serum with or without α-Naphthoflavone, data are presented as mean ± SEM (*n* = 5), statistical difference: ** *p* < 0.01 and *** *p* < 0.001. (**c**) H&E staining of mouse liver after treated α-Naphthoflavone, original scale: 10×. (**d**) The mRNA expression of mIl6, miNOS, mIl10 and mIl-1β, results are indicated as means ± SEM (*n* = 5), statistical difference: * *p* < 0.05, ** *p* < 0.01 and *** *p* < 0.001.

The Cyp1a2 inhibitor (α-Naphthoflavone) was used to verify the effect of Cyp1a2 in relation to Rut aggravating APAP-mediated hepatotoxicity. The levels of ALT and AST, as well as the H&E staining, showed no significant pathological changes in mice liver when comparing the α-Naphthoflavone group with the control group, indicating that the inhibitor itself did not influence the results (Figure 3a,c). Compared to the control group, APAP significantly increased the concentrations of ALT and AST. When 80 mg/kg Rut was administered to mice in combination with APAP, these levels increased further. However, α-Naphthoflavone reversed these abnormal increases very significantly.

The histologic observations revealed that Rut exacerbated the hepatic damage in the APAP-administered mice, showing extensive focal necrosis, cell membrane damage, nuclear shrinkage, and significant inflammatory cell infiltration. In contrast, α-Naphthoflavone significantly alleviated these symptoms (Figure 3c). The results showed that the inhibition of CYP1A2 activity by α-Naphthoflavone ameliorated the liver injury caused by Rut and APAP, implying that CYP1A2 plays a critical role in Rut-aggravated APAP-mediated hepatotoxicity. 

#### 3.2.2. The Expression of Pro-Inflammatory Cytokines

The pro-inflammatory cytokines, including Il6, Il-1β and iNOS, as well as the anti-inflammatory cytokine, Il10, could directly reflect the extent of the liver damage. In this study, the mRNA expression levels of Il6, Il-1β, iNOS and Il-10 in mice liver increased in the APAP group, and Rut further upregulated the levels of these factors. However, treatment with α-Naphthoflavone significantly decreased the mRNA levels of these cytokines (Figure 3d). These findings indicated that Rut could aggravate APAP-induced liver inflammation via upregulating the mRNA expression of pro-inflammatory cytokines, and it is also related to the Cyp1a2 expression [26].

### 3.3. Rut Aggravated the APAP-Induced Acute Liver Injury In Vitro

#### 3.3.1. Hepatocyte Injury Model In Vitro and Drug Concentration

**Figure 4:**
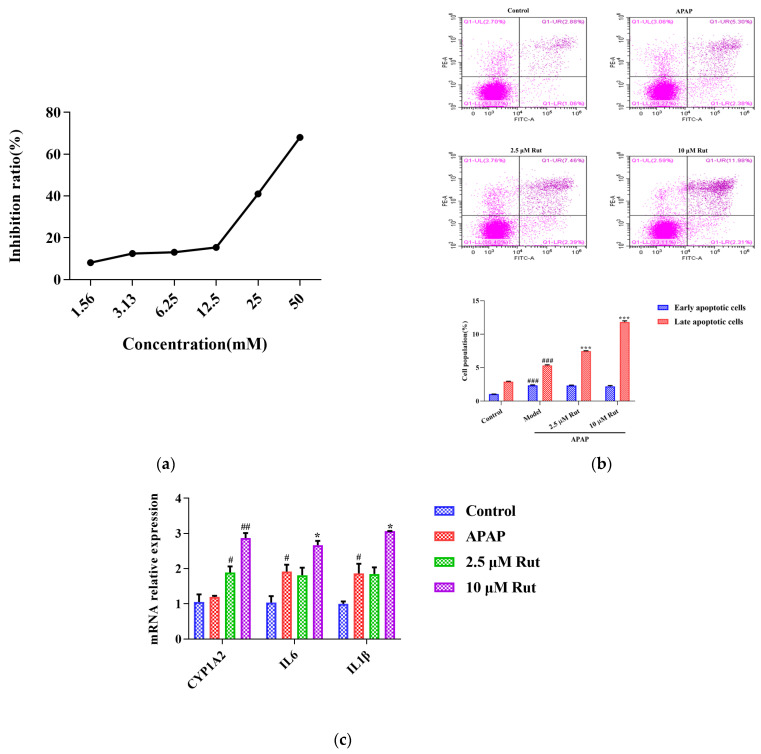
Rut aggravated the APAP-induced acute liver injury in vitro. (**a**) The relationship between the APAP concentration and L02 cell inhibition rate, 30 mM APAP is a suitable concentration for the model. (**b**) Apoptosis was detected by flow cytometry and date analysis, values are expressed as mean ± SEM (*n* = 3), statistical difference: compared with control, ^###^ *p* < 0.001; with APAP group *** *p* < 0.001. (**c**) The mRNA expression of CYP1A2, IL6 and IL-1β, values are expressed as mean ± SEM (*n* = 3), statistical difference: compared with control, ^#^ *p* < 0.05 and ^##^ *p* < 0.01; with APAP group * *p* < 0.05.

The CCK-8 assay was conducted to determine the appropriate concentrations and dosages of APAP and Rut for modeling. According to the IC50 value, 30 mM of APAP increased the cell inhibition ratio to around 50% after being incubated with L02 cells for 6 h (Figure 4a). Rut showed significant cytotoxicity at a concentration of 20 μM, while the cell viability remained above 98% at 10 μM after 24 h of incubation. Based on these results, a simulated injury model was constructed with L02 cells co-cultured with 30 mM APAP for 6 h. Cells cocultured with 2.5 μM and 10 μM Rut for 24 h were used as the drug concentrations in the following experiments.

#### 3.3.2. Rut Upregulates the Apoptosis Level of APAP-Induced Cells Injury

As shown in Figure 4b, flow cytometry analysis was used to evaluate whether Rut could exacerbate the apoptotic effect of APAP in L02 cells in vitro. The Annexin V/PI apoptosis assay revealed that the rates of the late stages of apoptosis in 40 mg/kg Rut + APAP group and 80 mg/kg Rut + APAP group were 7.46 (*p* < 0.01) and 11.98 (*p* < 0.01), respectively, which were significantly higher than in the APAP group. The results indicated that Rut increased the level of apoptosis in APAP-mediated cell injury.

#### 3.3.3. Rut Upregulates the mRNA Expression of CYP1A2 and Pro-Inflammatory Factors In Vitro

To further evaluate whether CYP1A2 was involved in Rut exacerbating the APAP-induced cell injury, we measured the mRNA expression of CYP1A2, along with several pro-inflammatory factors. The result revealed that the mRNA expression of CYP1A2 in the APAP group did not significantly change compared to the blank group, indicating that APAP did not affect the expression level of CYP1A2. However, CYP1A2 was significantly upregulated in both the 2.5 and 10 μM Rut groups. The RT-PCR results of the pro-inflammatory factors showed that 10 μM Rut could remarkably upregulate the mRNA expression of Il6 and Il1β, while there was no significant difference between the 2.5 μM Rut group and the APAP group (Figure 4c). According to these results, CYP1A2 was involved in the process of Rut aggravating the APAP-mediated cells injury, which is consistent with the in vivo findings.

## 4. Discussion

The combination of traditional Chinese medicine (TCM) with synthetic drugs is a common clinical practice, which is increasingly popular due to the enhanced therapeutic effects achieved through multi-drug regimens. However, the complexity of TCM compositions can lead to adverse reactions when used in conjunction with synthetic drugs. Therefore, it is essential to conduct a comprehensive investigation into the toxic components of TCM, the potential toxic interactions with synthetic drugs, the metabolic enzyme systems involved in these reactions, and the underlying metabolic mechanisms. This study focuses on how Rut, a component of TCM, exacerbates APAP-induced acute liver damage by inducing the drug-metabolizing enzyme CYP1A2. The findings underscore the importance of understanding these drug interactions and their metabolic mechanisms to ensure the safe and effective use of combined TCM and synthetic drug therapies. 

Hepatotoxicity can result from various factors, including excessive alcohol consumption, drugs, and viral infections. Among these, drug-induced toxicity is a major concern, with APAP overdose being the most common cause of acute liver injury. 

APAP is commonly used as an antipyretic and analgesic, and it generally has few side effects at therapeutic doses. However, an overdose of APAP can lead to liver injury beginning as early as 3 h post-administration and progressing to severe hepatocyte death within 24 h in mouse models [27,28,29,30]. CYP2E1 and CYP1A2 are the primary cytochrome P450 enzymes responsible for metabolizing APAP into its metabolite, NAPQI. Elevated levels of NAPQI deplete GSH, leading to the formation of NAPQI-GSH adducts. This process triggers mitochondrial oxidative stress and ultimately causes acute liver inflammation [31]. 

APAP overdose can cause serious hepatic injury, as characterized by high serum level of ALT/AST, which are indicators of significant hepatotoxicity [32,33]. Previous reports have suggested that Rut pretreatment can ameliorate hepatocyte damage by inhibiting the expression of CYP2E1, reducing ALT/AST release and decreasing GSH consumption [34,35,36]. In contrast to these findings, our study demonstrated that Rut pretreatment of mice and L02 cells could exacerbate the APAP-mediated acute liver injury. This exacerbation occurs through the upregulation of both the mRNA and protein expressions of CYP1A2, which enhances the formation of the toxic metabolite NAPQI from APAP. Our results indicate that while Rut has previously been reported to mitigate liver damage under certain conditions, it can also aggravate APAP-induced acute liver injury by modulating CYP1A2 expression and promoting the toxicity of APAP. 

Liver damage is always associated with the overexpression of pro-inflammatory factors, while a reduction in these factors is often related to the protection or restoration of hepatic function. Therefore, the inflammatory response is a crucial aspect of the mechanism underlying hepatotoxicity caused by APAP [27,37]. The APAP metabolite NAPQI can activate pro-inflammatory cytokines, such as IL-1β, IL-6, TNF-α, COX-2, and iNOS, leading to a significant inflammatory response [37]. In our study, we found that Rut exacerbates APAP-induced liver inflammation by further increasing the expression of Il-1β, Il-6, and iNOS, both in vivo and in vitro. These results suggest that Rut upregulates pro-inflammatory factors and aggravates APAP-induced liver inflammation. This process is linked to the upregulation of CYP1A2, which enhances the production of NAPQI and thereby intensifies the inflammatory response. A piece of more obvious evidence is that the exacerbation of APAP-induced hepatotoxicity by RUT can be mitigated by the CYP1A2-specific inhibitor α-Naphthoflavone. Unfortunately, the interaction of α-Naphthoflavone with APAP in hepatotoxicity was not involved in this study. However, the specific mechanisms through which Rut modulates CYP1A2 expression and influences inflammatory responses require further investigation to fully understand its role in APAP-induced liver injury and to explore the relationship between metabolic enzymes and inflammatory processes.

## 5. Conclusions

In summary, Rut exacerbates APAP-mediated liver damage by upregulating CYP1A2 and increasing the levels of pro-inflammatory factors. These results underscore the importance of carefully considering the dosage of Rut when used in combination with APAP. This study offers valuable insights for exploring the mechanisms of drug interactions in clinical practice, particularly in the context of combining TCM with synthetic pharmaceuticals.

## Data Availability

The data underlying this article will be shared on reasonable request to the corresponding author.

## Supplementary Materials

The following supporting information can be downloaded at https://www.mdpi.com/article/10.3390/toxics12070515/s1. Table S1: The primer for the mouse; Table S2: The primer for the human.
