# Supplementary material for: Rutaecarpine Aggravates Acetaminophen-Induced Acute Liver Injury by Inducing CYP1A2
Source: Toxics. 2024 Jul 18;12(7):515. doi: 10.3390/toxics12070515 (PMC11280960; doi:10.3390/toxics12070515)
Supplement: Supplementary file 1 [file toxics-12-00515-s001.zip › toxics-3060883-supplementary.pdf]

Table S1. The primer of mouse

Primer	Fw (5′~3′)	Re (5′~3′)
mβ-actin	TTCCTTCTTGGGTATGGAATCCTG	GAGGTCTTTACGGATGTCAACG
mCyp2e1	CGTTGCCTTGCTTGTCTGGA	AAGAAAGGAATTGGGAAAGGTCC
mCyp1a2	AGTACATCTCCTTAGCCCCAG	GGTCCGGGTGGATTCTTCAG
mIl6	TAGTCCTTCCTACCCCAATTTCC	TTGGTCCTTAGCCACTCCTTC
mIl1β	GCCACCTTTTGACAGTGATGAG	GACAGCCCAGGTCAAAGGTT
mIl10	GCTCTTACTGACTGGCATGAG	CGCAGCTCTAGGAGCATGTG
miNos	GTTCTCAGCCCAACAATACAAGA	GTGGACGGGTCGATGTCAC

Table S2. The primer of human

Primer	Fw (5′~3′)	Re (5′~3′)
GADPH	GACCCCTTCATTGACCTCAAC	CATACCAGGAAATGAGCTTG
Human CYP1A2	CTGGAGACCTTCCGACACT	ACCTGCCACTGGTTTACG
Human IL6	AGACAGCCACTCACCTCTTC	AGTGCCTCTTTGCTGCTTTC
Human IL1β	AGAAGTACCTGAGCTCGCCA	CTGGAAGGAGCACTTCATCTGT
